# Dual roles of renal tubular mitochondrial Akt1 in protecting against diabetic nephropathy and improving body glucose metabolism

**DOI:** 10.1530/JOE-26-0143

**Published:** 2026-09-15

**Authors:** Esam S B Salem, Albert Ta, Elizabeth Bloom-Saldana, Patrick T Fueger, Ping H Wang

**Affiliations:** ^1^ Department of Diabetes, Endocrinology and Metabolism, City of Hope National Medical Center, Duarte, California, USA; ^2^ Department of Molecular and Cellular Endocrinology, City of Hope, Duarte, California, USA; ^3^ Comprehensive Metabolic Phenotyping Core, City of Hope, Duarte, California, USA

**Keywords:** mitochondrial Akt1, diabetic nephropathy, albuminuria, renal proximal tubules, TGFβ, αSMA

## Abstract

Diabetic nephropathy (DN) is a leading cause of chronic kidney failure. We hypothesized that mitochondrial Akt1 dysfunction in renal proximal tubules plays a pathogenic role in DN development and that its activation may reverse DN progression. To study this signaling pathway, we generated a transgenic mouse model harboring a renal tubule-specific, tamoxifen-inducible, mitochondria-targeted constitutively active Akt1 (KMCAKT). Type 2 diabetes was induced by a high-fat, high-fructose diet (HFFD) for 40 weeks. Renal histology and function were evaluated, and glucose metabolism was assessed using dynamic glucose testing. We found that HFFD feeding resulted in the development of DN in control non-induced KMCAKT mice, whereas KMCAKT mice with constitutively active mitochondrial Akt1, induced by tamoxifen injection (TAM), exhibited a significant improvement in kidney dysfunction and histology. Urinary albumin, fasting plasma BUN levels, fibrosis, and Jablonski scores were all markedly improved in HFFD-TAM-KMCAKT mice compared with controls, while levels of α-smooth muscle actin (αSMA) and transforming growth factor-β1 (TGFβ1) were significantly reduced. HFFD-TAM-KMCAKT mice exhibited lower fasting blood glucose and improved oral glucose tolerance, while basal and stimulated insulin levels were higher, along with increased beta cell mass and insulin secretion (HOMA-β) compared with controls. Hyperglycemic clamp studies confirmed increased insulin secretion in HFFD-TAM-KMCAKT mice. We conclude that tubular mitochondrial Akt1 plays a key role in DN progression. Restoring tubular mitochondrial Akt1 signaling may represent a novel approach to reverse the development of chronic kidney disease. We also identified previously unrecognized metabolic cross talk between renal tubular mitochondrial Akt1 and pancreatic insulin secretion that may modulate systemic glucose homeostasis.

## Introduction

Chronic hyperglycemia can lead to the development of diabetic nephropathy (DN). DN is a serious and common complication of diabetes and remains a major cause of chronic kidney disease (CKD) and end-stage renal disease (ESRD) worldwide (1, 2). Diabetic kidney disease is associated with a high risk of cardiovascular disease (CVD) and mortality (3). As the global prevalence of diabetes continues to rise (4), the incidence of DN is expected to increase, imposing a growing public health and socioeconomic burden (5, 6). Despite advances in diabetes management, a substantial proportion of patients still progress to DN, and rates of progression to ESRD remain high (7, 8). This problem underscores the urgent need to elucidate the molecular mechanisms driving DN progression and to identify novel molecular targets for the development of new therapeutic strategies.

Early DN can be diagnosed by persistent microalbuminuria, reflecting early glomerular injury, and a subsequent progressive decline in estimated glomerular filtration rate (eGFR), indicating worsening renal function (1, 9). Histological changes are characterized by mesangial expansion due to the accumulation of extracellular matrix proteins, podocyte injury and loss, thickening of the glomerular basement membrane, nodular or diffuse glomerulosclerosis, and tubulointerstitial fibrosis (10). Together, these structural and functional changes reflect the complex pathogenesis of DN, involving metabolic, hemodynamic, and inflammatory mechanisms that collectively contribute to kidney injury.

Mitochondria play a pivotal role in maintaining renal homeostasis, and their dysfunction is increasingly recognized as a key element in the development of CKD (11). In experimental DN, alterations in renal mitochondrial bioenergetics and structural dynamics occur early, preceding the development of albuminuria and histological abnormalities (12). Hyperglycemia promotes excessive mitochondrial reactive oxygen species (ROS) production, oxidative stress, and mitochondrial dysfunction (13, 14). This diabetes-induced mitochondrial dysfunction disrupts fatty acid oxidation in renal tubular epithelial cells, leading to lipotoxicity and tubular injury (15, 16). Concurrently, defects in mitochondrial quality control, characterized by impaired mitophagy and reduced biogenesis, further exacerbate mitochondrial dysfunction (17, 18, 19, 20). Additionally, increased mitochondrial DNA (mtDNA) release activates innate immune signaling pathways that amplify inflammation and accelerate DN progression (21). Consistent with these experimental findings, patients with DN exhibit reduced mitochondrial content and evidence of mitochondrial dysfunction (22).

Activation of Akt1, a serine/threonine protein kinase, is a critical step in insulin receptor cytosolic signaling pathways and is essential for maintaining mitochondrial function. Notably in renal tubules, insulin stimulation results in robust translocation of activated Akt1 from the cytosol to the mitochondria, resulting in a 12-fold increase in its phosphorylated active form within mitochondria (23). Our previous work demonstrated that activation of mitochondrial Akt1 in the heart attenuates diabetic cardiomyopathy and improves whole-body metabolism (24). Similar to the myocardium, the kidney has exceptionally high energy demands, particularly within renal proximal tubular cells, which contain the highest mitochondrial density among renal cell types (25). Given the emerging evidence supporting the involvement of the kidney in metabolic syndrome, we postulate that mitochondrial Akt1 activation in the kidney may play a similarly protective role in diabetic kidney. We have previously demonstrated that Akt1 translocation into renal tubular mitochondria mitigates acute kidney injury and limits the subsequent development of CKD following ischemia–reperfusion injury (26). In contrast, inhibition of tubular mitochondrial Akt1 led to loss of mitochondrial cristae, disruption of ATP synthase subunit assembly, and severe mitochondrial dysfunction (24).

In the present study, we sought to define the role of sustained tubular mitochondrial Akt1 signaling in an insulin-resistant model of DN and to determine how renal tubular mitochondria may influence whole-body glucose metabolism. To this end, we used an inducible, renal tubule-specific transgenic mouse model to selectively activate mitochondrial Akt1 signaling (KMCAKT) and demonstrated that proximal tubular mitochondrial Akt1 signaling confers protection against diet-induced DN and systemic metabolic dysregulation.

## Materials and methods

### Transgenic mouse model

Animal care and experiments were performed according to procedures approved by the animal care committees of the City of Hope. A mitochondria-targeting constitutively active Akt1 construct was generated using strategies based on the approach we described previously (26). To generate a constitutively active mitochondrial Akt1 (mcaAkt1), threonine T308 and serine S473 of Akt1 cDNA were mutated to glutamic acid to mimic phosphorylation. For mitochondrial targeting, the human cytochrome c oxidase subunit 8A sequence (NP_004065.1; MSVLTPLLLRGLTGSARRLPVPRAKIHSL) was added to the 5′ end of the Akt1 cDNA. A 6× His-tag was added at the 3′ end for the detection of the resultant protein. The mitochondria-targeting sequence is cleaved after mitochondrial import (24, 26).

**Figure 1:**
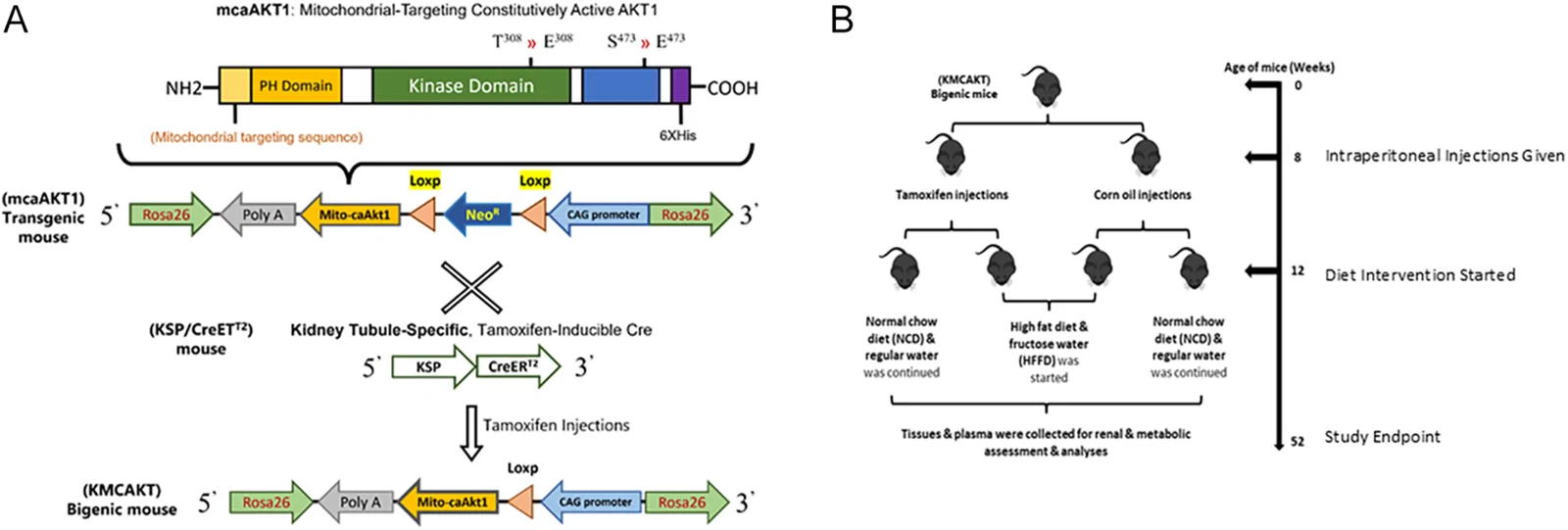
Bigenic mice (KMCAKT) for renal tubule-specific mitochondria-targeting constitutively active Akt1, and study design. (A) Mitochondrial-targeted constitutively active Akt1 (*mcaAkt1*) was engineered as described in the Materials and methods section. A 6× His tag was fused in frame to the C-terminus of Akt1 cDNA. Mice harboring *mcaAkt1* were crossed with KSP/CreER^T2^ mice to generate bi-transgenic mice (KMCAKT) for this study. (B) Eight-week-old KMCAKT mice were randomized into two groups – one injected with tamoxifen (TAM) to induce expression of mcaAkt1 and the other with corn oil (CO) as a vehicle control. Each group was then subdivided into either a normal chow diet (NCD) with regular water or a high-fat diet with fructose water (HFFD) starting at twelve weeks of age and maintained for up to 40 weeks. Tissues and plasma were collected for renal and metabolic analyses at the study endpoint.

We next cloned the mcaAkt1 construct into the ROSA26 locus of C57BL/6J mice. The coding sequence was first cloned into pCALNL-dsRed, a gift from Dr Constance Cepko (Addgene, 13769), after the removal of the Ds-Red coding sequence. In pCALNL, a CMV immediate early gene enhancer and chicken beta-actin gene promoter/intron and beta-globin polyA signal drive the expression of a coding sequence. To mediate targeting to the ROSA26 (Gt (ROSA)26Sor) locus, mcaAKT1-pCALNL was cloned into pROSA26-1, a gift from Dr Philippe Soriano (Addgene, 21714), with the CAG promoter oriented opposite to the ROSA26 RNA transcript. Expression of mcaAkt1 begins following Cre-mediated deletion of a floxed Neo-SV40-PolyA sequence between the promoter and the coding sequence. JM8.N4 ES cells (derived from C57BL/6NTac mice) were electroporated with linearized targeting constructs, and 32 G418-resistant clones per construct were screened for homologous recombination. Targeting efficiency was 53% for the mcaAkt1 construct. Correctly targeted ES cells were microinjected into C57BL/6J blastocysts, and the resulting male ROSA26-CAG-LNL-mcaAKT1 chimeras were bred with C57BL/6 mice to establish the lines used in this study (24, 26). Engineering of mES cells, genomic characterization, microinjection of blastocysts, and production of founder transgenic mice were carried out at the UC Irvine Transgenic Mouse Facility. To generate a transgenic mouse line with inducible overexpression of mcaAkt1 in the renal proximal tubular epithelial cells, ROSA26-CAG-LNL-mcaAkt mice were crossed with a well-studied Cre transgenic mouse strain, KSP-CreERT2 mice (Tg(Cdh16-Cre/ERT2)24Igr) on a congenic C57BL/6 background (purchased from The Jackson Laboratory), to generate KMCAKT mice (Fig. 1A).

Mice were kept in a temperature-controlled environment and fed with laboratory chow *ad libitum* (normal chow diet, NCD; Envigo Teklad, USA, Envigo 2020X). Cre-mediated recombination was achieved by administration of tamoxifen (TAM; 100 mg/kg body weight/day i.p.) for 5 days at 8 weeks of age. Corn oil (CO) was injected as a vehicle control (Fig. 1B). Expression of the transgene was shown specifically in the renal tubules in our previous study (26). One month after initial TAM or CO injection, the mice were subjected to high-fat chow and fructose drinking water as described below (Fig. 1B).

### Diet-induced diabetes

Mice were placed on a high-fat chow and fructose water diet (HFFD). High-fat chow contains 45% of calories from fat (Envigo Teklad, TD.08811). Cage water was replaced with a 30% fructose solution (Sigma, USA, F0127-5KG). The length of HFFD is specified per given experiment (Fig. 1B). Chow and water were replaced at two-week intervals. Male mice were used due to their susceptibility to diet-induced metabolic dysregulation and dysfunction, including insulin resistance, hyperglycemia, and DN.

### Body composition measurement

Body composition was measured using ^1^H magnetic resonance spectroscopy (Echo Medical Systems, USA, EchoMRI-100). After calibration of the apparatus, mice were introduced into a clean and transparent plastic cylinder and placed into the apparatus to determine fat content, lean mass, and total body water.

### Urine collection

Mice were placed individually in metabolic cages with free access to food and water for 24-h urine collection. Protease inhibitors (Roche, Switzerland, 4693116001) were added to prevent protein degradation. Urine samples were collected in two steps every 12 h and kept at 4°C until the 24-h collection was completed. Samples were centrifuged at 3,000 *g* for 5 min at 4°C to remove cellular debris, and supernatants were aliquoted and stored at −80°C until use.

### Western blot analysis

Kidney tissues were homogenized on ice using cOmplete Lysis-M EDTA-free buffer (Roche, 4719964001) containing 2.5 mmol/L PMSF and protease inhibitors. Tissue homogenates were centrifuged at 10,000 *g* for 10 min at 4°C to remove cellular debris. Kidney protein lysates (15 μg per lane) were separated on 10% SDS-PAGE and electroblotted to polyvinylidene fluoride membranes (Millipore, USA). Membranes were incubated overnight at 4°C with the following specific primary antibodies: mouse anti-TGFβ1 (Santa Cruz Biotechnology, USA, 130348, 1:1,000), mouse anti-αSMA (Invitrogen, USA, MA106110, 1:1,000), or rabbit anti-GAPDH (Cell Signaling Technology, USA, 2118, 1:1,000), followed by incubation with horseradish peroxidase-conjugated goat anti-mouse (Invitrogen, PI31432, 1:5,000) or goat anti-rabbit (Invitrogen, PI31466, 1:5,000) secondary antibodies. Signals were detected using a SuperSignal chemiluminescent substrate (Thermo Scientific, USA, 34579) and visualized using a Syngene G:BOX imaging system.

### Kidney histology and immunohistochemistry

Kidneys were collected from mice anesthetized with ketamine/xylazine (100:8 mg/kg) and perfused with PBS followed by 4% paraformaldehyde. Tissues were fixed in 4% PFA overnight at 4°C, embedded in paraffin, sectioned, and stained with periodic acid–Schiff and Masson’s trichrome. Images were obtained using a Zeiss LSM700 microscope.

### Urinary albumin assay

Urinary albumin excretion was determined using a mouse albumin ELISA kit (Fortis Life Sciences, USA, E99-134) in accordance with the manufacturer's instructions. Briefly, a 96-well plate coated with goat anti-mouse albumin antibody was incubated with diluted albumin standards and urine samples. After sample binding, unbound proteins were washed off, and a biotinylated detection antibody was added to bind the captured albumin. A streptavidin-conjugated horseradish peroxidase was then added to catalyze a colorimetric reaction with the chromogenic substrate TMB. The reaction produced a blue product, which turned yellow when the reaction was terminated by the addition of sulfuric acid (2 N H_2_SO_4_) solution. Absorbance at 450 nm was measured using a Tecan Spark plate reader.

### Urinary and plasma creatinine assay

Urinary and plasma creatinine levels were measured by ELISA (Quidel, USA, MicroVue^TM^ Creatinine EIA, 8009). Urine and plasma samples and standards were diluted with distilled water (1:40) and then loaded into a 96-well plate, followed by the addition of a color reagent. After a 30-min incubation, measurements of optical density were taken at 450 nm using a Tecan Spark plate reader.

### Plasma BUN, TGFβ1, insulin, and lipid measurements

Blood samples were collected in ice-chilled heparinized tubes. Blood was centrifuged at 10,000 *g* for 10 min at 4°C. Plasma was separated, aliquoted, and stored at −80°C. Plasma samples were analyzed for determining the levels of BUN (Urea Nitrogen Colorimetric Detection Kit K024-H1, Arbor Assays, USA), TGFβ1 (mouse/human TGFβ1 Quantikine ELISA Kit DB100C, R&D Systems, USA), insulin (Ultra-Sensitive Mouse Insulin ELISA Kit, 2nd Gen 62100, Crystal Chem, USA), total cholesterol (#Infinity^TM^ Cholesterol Kit 812912, Thermo Scientific), and triglycerides (Infinity^TM^ Triglycerides Kit 707440, Thermo Scientific) according to the manufacturer’s instructions.

### Measurement of blood glucose levels and oral glucose tolerance test (OGTT)

Blood glucose concentration was determined using a glucometer (FreeStyle Lite, Abbott, USA) and FreeStyle blood glucose test strips. A gentle cut was made at the tip of the mouse’s tail to draw venous blood samples for measurement. OGTT was performed by oral glucose administration (2 g/kg) following food withdrawal for 6 h. Values were recorded and expressed in milligrams per deciliter.

### Assessment of insulin resistance and pancreatic β-cell function

Fasting blood glucose and plasma insulin levels were measured after a 6-h fast. Insulin resistance was calculated using the homeostasis model assessment of insulin resistance (HOMA-IR). Pancreatic β-cell function was analyzed with the homeostasis model assessment of β-cell function (HOMA-β) (27).

#### Pancreatic tissue quantification

For immunofluorescence imaging of pancreatic tissue, sections were deparaffinized and incubated in 10 mM sodium citrate (pH 8.5) at 95°C for 60 min. Sections were then blocked in 10% normal goat serum and incubated with rabbit anti-insulin (EPR17359, Abcam, UK) and mouse anti-glucagon (67286, Proteintech, USA), diluted in PBS containing (0.1% Triton X-100) overnight at 4°C. The sections were then washed with PBS three times and incubated with goat anti-rabbit Alexa Fluor^TM^ 488 (H+L) (A11008, Invitrogen) and goat anti-mouse Alexa Fluor^TM^ 647 (H+L) (A21235, Invitrogen). Slides were allowed to air-dry and coverslipped using ProLong^TM^ Gold antifade reagent (P10144, Invitrogen). Images were obtained using a Zeiss LSM700 microscope. Quantification was performed with QuPath 0.5.1. Absolute β-cell mass was determined by multiplying the percent of insulin-positive area/pancreatic area (from four consecutive levels of pancreatic tissue 150 μm apart) with total pancreas weight (28). Similarly, ɑ-cell mass was determined by quantifying glucagon-positive area of the pancreatic sections and used to calculate alpha cell-to-beta cell ratios.

### Hyperglycemic clamp

A hyperglycemic clamp was performed in 5-h-fasted, chronically catheterized, conscious mice at the City of Hope Beckman Research Institute’s Comprehensive Metabolic Phenotyping Core. Briefly, carotid artery and jugular vein catheters were implanted for blood sampling and infusion 4 days prior to the study, as described in a previous study (29). Only mice that returned to within 10% of pre-surgical body weight were studied. Arterial blood glucose was measured every 10 min (∼5 μL whole blood), and a continuous infusion of 50% dextrose was adjusted as necessary to maintain hyperglycemia (250–300 mg/dL). Blood samples (∼100 μL each) were collected at −15, −5, 5, 10, 15, 30, 60, 90, and 120 min to measure plasma insulin (Mouse Insulin ELISA Kit 10-1247-01, Mercodia, Sweden) and C-peptide (Mouse C-Peptide ELISA Kit 90050, Crystal Chem, Sweden) according to the manufacturer’s instructions.

### Statistical analysis

Statistical analysis was performed with GraphPad Prism (10.0). All data are presented as mean ± SEM. An unpaired Student’s t-test was used to evaluate the differences between two groups. For comparisons between more than two groups, one-way ANOVA was used. The differences in hyperglycemic clamp measures (blood glucose level, glucose infusion rate, plasma insulin, and C-peptide) were assessed by two-way repeated measures ANOVA. A value of *P* < 0.05 was considered statistically significant. If a significant difference was identified, Tukey’s multiple comparison test was performed.

## Results

### Activation of tubular mitochondrial Akt1 protected against development of HFFD-induced nephropathy

KMCAKT mice were injected with tamoxifen (TAM) intraperitoneally to induce Cre-mediated recombination and transgene activation; corn oil (CO) injection was used as a vehicle control (Fig. 1A and B). To establish a high-fat/high-fructose diet (HFFD)-induced DN model, twelve-week-old male CO-KMCAKT and TAM-KMCAKT mice were placed on a HFFD diet for 40 weeks, while control diet mice were fed standard chow and regular water (NCD) (Fig. 1B), as described in detail in the Materials and methods section. Body composition was analyzed to quantify adiposity in these mice. HFFD-fed mice exhibited increased body fat and elevated fasting plasma cholesterol without changes in food intake, water intake, or total body water compared with NCD-fed mice (Table 1). Collectively, these findings reflect increased adiposity, a hallmark of diet-induced obesity, which contributes to insulin resistance, hyperglycemia, and metabolic stress despite unchanged daily food and water intake.

**Table 1:** Changes in metabolic and plasma parameters of KMCAKT mice injected with corn oil (CO) or tamoxifen (TAM) and fed with normal chow diet (NCD) or high-fat and high-fructose diet (HFFD).

Parameter	NCD-CO	NCD-TAM	HFFD-CO	HFFD-TAM
Age (weeks)	32	32	32	32
Duration of HFFD (weeks)	20	20	20	20
Group size (*n*)	8	8	6	6
Total body weight (g)	38.56 ± 1.29	41.21 ± 2.29	61.85 ± 1.71****	59.80 ± 2.75^ns^
Absolute body fat (g)	7.47 ± 0.63	11.74 ± 1.94	29.18 ± 0.74****	27.11 ± 1.90^ns^
Body lean mass (g)	30.12 ± 0.99	29.00 ± 0.53	33.65 ± 1.05*	33.43 ± 1.11^ns^
Body total water (g)	25.92 ± 0.75	24.67 ± 0.37	27.03 ± 0.74	27.36 ± 0.83^ns^
Food intake (g/day)	4.54 ± 0.28	3.95 ± 0.34	4.66 ± 1.01	3.15 ± 0.12^ns^
Water intake (g/day)	4.76 ± 0.34	3.61 ± 0.48	4.86 ± 0.29	4.40 ± 0.34^ns^
Plasma cholesterol (mg/mL)	49.45 ± 1.76	52.85 ± 2.58	143.1 ± 6.76****	129.22 ± 4.72^ns^
Plasma triglycerides (mg/mL)	72.33 ± 1.71	69.17 ± 2.36	65.47 ± 0.84*	67.23 ± 1.99^ns^

Values are mean ± SEM. Statistically significant: *P < 0.05, **P < 0.005, ***P < 0.0005, or ****P < 0.0001 vs. age-matched NCD-CO-KMCAKT mice; ns, not significant vs. age-matched HFFD-CO-KMCAKT mice.

**Figure 2:**
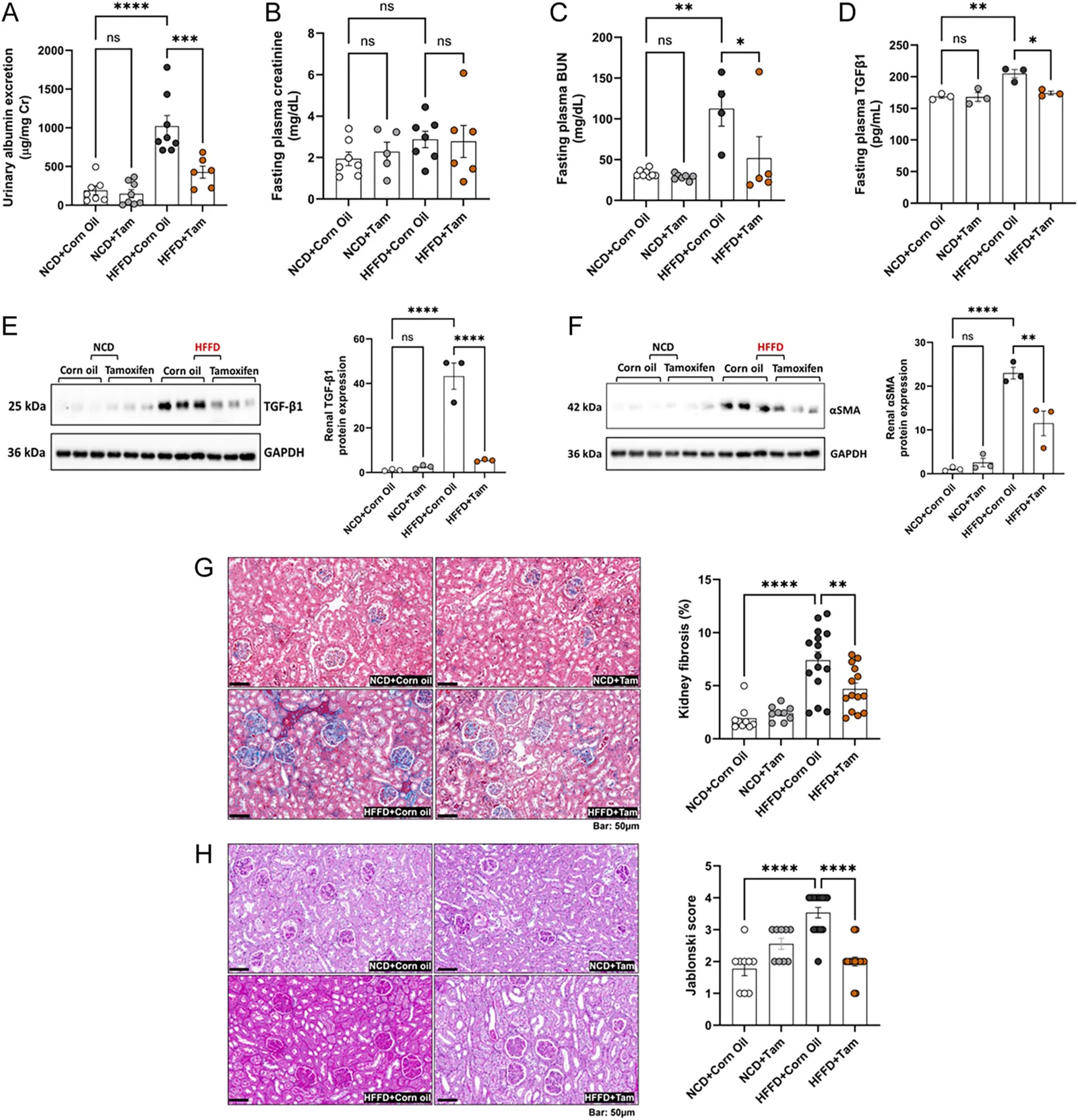
Activation of renal tubular mitochondrial Akt1 attenuates diet-induced diabetic nephropathy. (A) Significantly higher urinary albumin levels were observed in the HFFD-fed KMCAKT mice injected with corn oil (HFFD-CO-KMCAKT), which were attenuated in HFFD-fed KMCAKT mice with mitochondrial Akt1 activation induced by tamoxifen injection (HFFD-TAM-KMCAKT). (B) Measurement of fasting plasma creatinine showed no significant changes among the four groups. (C) Fasting plasma BUN was elevated by HFFD and further elevated in HFFD-CO-KMCAKT mice compared with HFFD-TAM-KMCAKT mice. (D) Fasting plasma TGFβ1 were significantly higher in HFFD-CO-KMCAKT mice compared with HFFD-TAM-KMCAKT mice. (E, F) Western blot analyses confirmed increased protein expression of TGFβ1 and αSMA in the kidneys of HFFD-CO-KMCAKT mice after 40 weeks of HFFD, which was reduced by mitochondrial Akt1 activation in HFFD-TAM-KMCAKT mice. (G, H) Representative microscopic images of Masson’s trichrome- and PAS-stained kidney sections showed increased fibrosis, and Jablonski scores for renal injuries were elevated in HFFD-CO-KMCAKT mice after 40 weeks of HFFD. These effects were ameliorated by activation of mitochondrial Akt1 in HFFD-TAM-KMCAKT mice. Data are shown as mean ± SEM (*n* = 3–8). Statistical significance was determined by one-way ANOVA, *P < 0.05, **P < 0.005, ***P < 0.0005, or ****P < 0.0001 versus the control group. BUN, blood urea nitrogen; PAS, periodic acid–Schiff; ns, not significant.

Compared with the NCD-fed KMCKAT mice, HFFD-fed KMCAKT mice exhibited increased urinary albumin excretion, whereas creatinine levels remained normal, indicating early nephropathy (Fig. 2A and B). Blood urea nitrogen (BUN) levels were also elevated following HFFD feeding (Fig. 2C). In HFFD-TAM-KMCAKT mice, elevations in albumin excretion and BUN were significantly attenuated compared with HFFD-CO-KMCAKT mice, indicating improved renal function (Fig. 2A and C). Transforming growth factor β1 (TGFβ1), which plays a pathogenic role in CKD progression, was increased in HFFD-CO-KMCAKT mice and reduced in HFFD-TAM-KMCAKT mice (Fig. 2D and E). Similarly, renal αSMA content, a marker of epithelial-to-mesenchymal transition, was increased in HFFD-CO-KMCAKT mice and decreased in HFFD-TAM-KMCAKT mice (Fig. 2F). Kidney fibrosis and Jablonski scores were analyzed to confirm histological changes. Masson’s trichrome staining revealed a significant increase in fibrosis and glomerulosclerosis in diabetic HFFD-CO-KMCAKT mice compared with age-matched NCD-CO-KMCAKT mice. These results confirmed that induction of type 2 diabetes was accompanied by kidney injury characteristic of DN following HFFD feeding. Activation of mitochondrial Akt1 in TAM-HFFD-KMCAKT mice attenuated diet-induced renal fibrosis (Fig. 2G). As with the changes observed in renal fibrosis, activation of tubular mitochondrial Akt1 also improved the Jablonski score in HFFD-TAM-KMCAKT mice (Fig. 2H), which recapitulates an overall improvement in renal function with tubular mitochondrial Akt1 activation.

### Activation of tubular mitochondrial Akt1 improved glucose metabolism in diabetic mice

**Figure 3:**
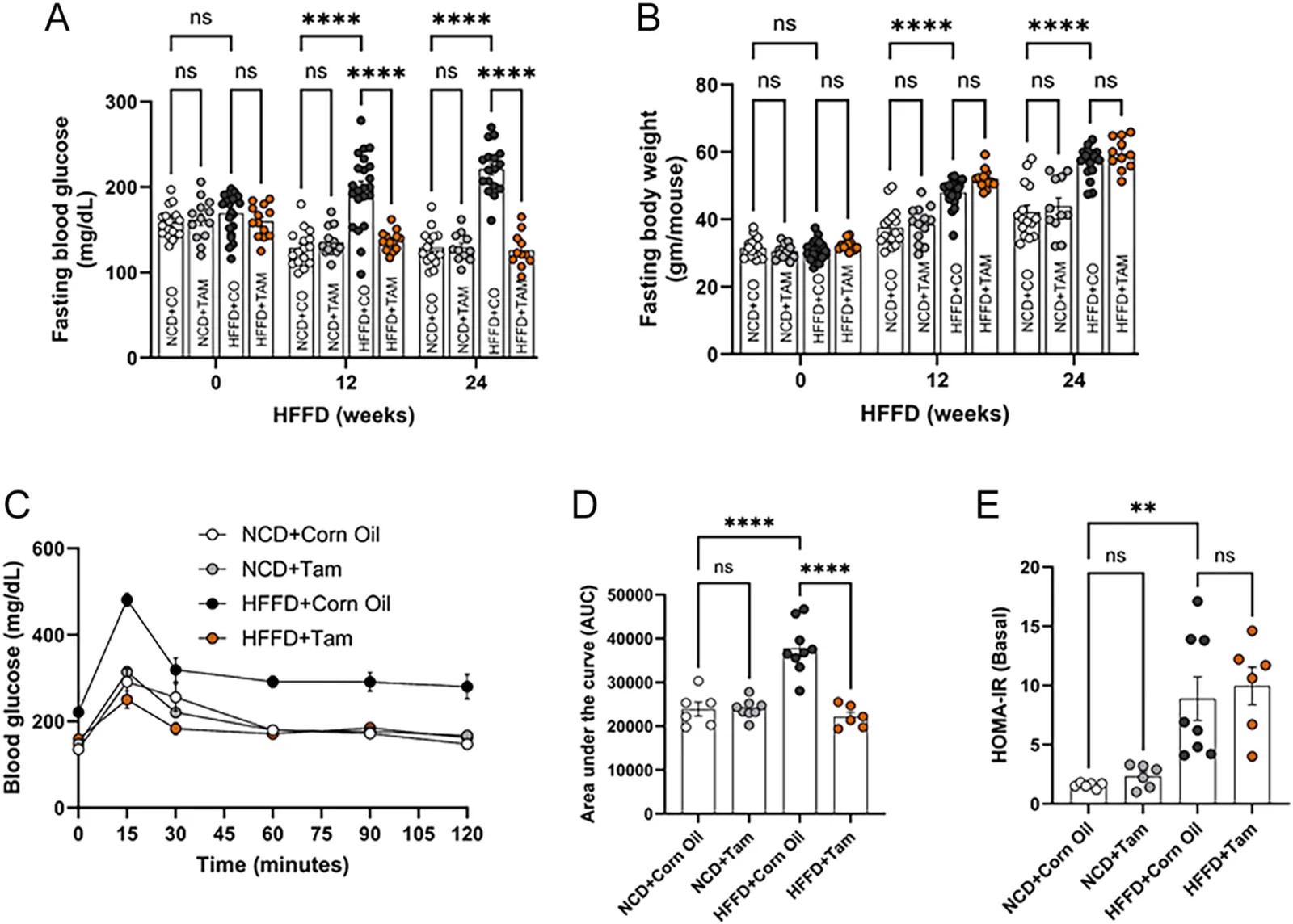
Renal tubule mitochondrial Akt1 activation improves diet-induced hyperglycemia and glucose intolerance without changes in body weight or insulin sensitivity. (A) Fasting blood glucose levels were comparable among the four groups of mice before HFFD feeding (week 0) but were significantly higher in HFFD-CO-KMCAKT mice compared with NCD-fed groups and HFFD-TAM-KMCAKT mice at weeks 12 and 24. (B) Body weights were comparable among the four groups of mice at week 0 but were significantly higher in the HFFD-fed groups compared with NCD-fed groups at weeks 12 and 24. (C) Oral glucose tolerance tests were performed in mice fed HFFD or NCD for 20 weeks. Blood glucose levels were significantly elevated in HFFD-CO-KMCAKT compared with HFFD-TAM-KMCAKT mice. (D) The AUC was higher in HFFD-CO-KMCAKT mice compared with NCD-fed groups and HFFD-TAM-KMCAKT mice. (E) Insulin resistance, assessed by HOMA-IR, was significantly elevated in HFFD-fed mice compared with NCD-fed mice and was unchanged between TAM- and CO-injected mice under HFFD. Data are shown as mean ± SEM (*n* = 6–22). Statistical significance was determined by one-way ANOVA, *P < 0.05, **P < 0.005, ***P < 0.0005, or ****P < 0.0001 versus the control group. AUC, area under the curve; HOMA-IR, homeostasis model assessment of insulin resistance; OGTT, oral glucose tolerance test.

At baseline, there was no significant difference in fasting blood glucose or body weight among the four groups of mice (Fig. 3A and B). HFFD-CO-KMCAKT mice exhibited progressively higher fasting blood glucose levels and body weight (Fig. 3A and B). Activation of tubular mitochondrial Akt1 in HFFD-TAM-KMCAKT mice improved fasting hyperglycemia but did not change body weight compared with HFFD-CO-KMCAKT mice (Fig. 3A and B).

To further investigate glucose homeostasis, oral glucose tolerance tests (OGTTs) were conducted to analyze dynamic glucose changes. HFFD-CO-KMCAKT mice showed impaired glucose tolerance compared with NCD-CO-KMCAKT mice; both peak blood glucose levels and the area under the curve (AUC) were increased (Fig. 3C and D). Glucose intolerance was ameliorated in HFFD-TAM-KMCAKT mice, indicating that activation of tubular mitochondrial Akt1 improved whole-body glucose metabolism. Because insulin action can be modulated by insulin sensitivity, insulin resistance was calculated. The results showed a significant increase in insulin resistance after HFFD feeding as expected, but HOMA-IR was not different between HFFD-CO-KMCAKT mice and HFFD-TAM-KMCAKT mice (Fig. 3E). This finding suggests that the glucose-lowering effects of tubular mitochondrial Akt1 were not due to changes in insulin sensitivity, but rather likely due to increased insulin secretion.

### Renal tubular mitochondrial Akt1 enhanced insulin secretion associated with increased β-cell mass in diet-induced diabetes

**Figure 4:**
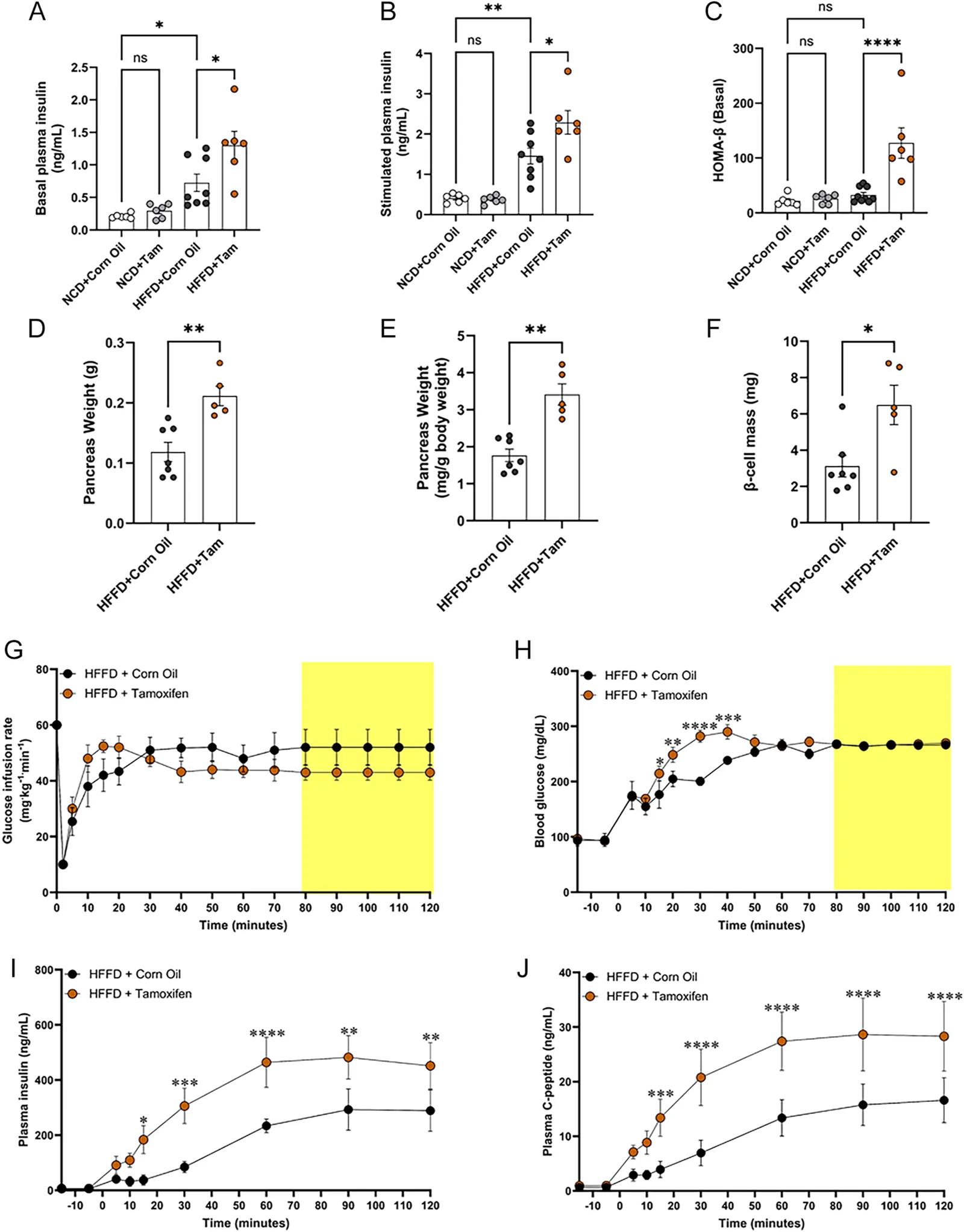
Tubular mitochondrial Akt1 activation enhances insulin secretion in diet-induced obesity and diabetes. (A) Basal plasma insulin levels after a 6-h daytime fast and (B) plasma insulin levels 15 min after oral glucose administration in mice fed NCD or HFFD for 24 weeks were measured. Basal and glucose-stimulated plasma insulin levels in HFFD-CO-KMCAKT mice were higher than in NCD-fed groups but were lower compared with HFFD-TAM-KMCAKT mice. (C) HOMA-β levels were higher in HFFD-TAM-KMCAKT mice compared with HFFD-CO-KMCAKT mice. (D, E) Absolute pancreas weight and pancreas weight normalized to body weight were higher in HFFD-TAM-KMCAKT mice compared with HFFD-CO-KMCAKT mice. (F) Absolute β-cell mass was higher in HFFD-TAM-KMCAKT mice compared with HFFD-CO-KMCAKT mice. (G, H) During the hyperglycemic clamp, mice received a glucose infusion to maintain blood glucose levels at ∼280 mg/dL for 2 h. (I, J) Plasma insulin and C-peptide levels were measured over time and were significantly higher in HFFD-TAM-KMCAKT mice compared with HFFD-CO-KMCAKT mice. Data are shown as mean ± SEM (*n* = 5–8). Statistical significance was determined by an unpaired Student’s *t*-test, one-way ANOVA, and two-way ANOVA, *P < 0.05, **P < 0.005, ***P < 0.0005, or ****P < 0.0001 versus the control group. HOMA-β, homeostasis model assessment of β-cell function.

Basal and postprandial insulin levels were measured during OGTTs to further characterize insulin dynamics. As anticipated, basal and postprandial insulin levels were increased in HFFD-fed mice compared with normal chow-fed mice. Moreover, basal and postprandial insulin levels were further increased in HFFD-TAM-KMCAKT mice compared with HFFD-CO-KMCAKT mice (Fig. 4A and B). Calculation of the HOMA-β index, a measure of insulin secretion, showed significant augmentation in HFFD-TAM-KMCAKT mice (Fig. 4C). Pancreas weight was greater in HFFD-TAM-KMCAKT compared with HFFD-CO-KMCAKT (Fig. 4D and E). In turn, β-cell mass, quantified by percentage area of insulin-positive staining of pancreatic tissue sections multiplied by pancreas weight, was also higher in HFFD-TAM-KMCAKT mice compared with HFFD-CO-KMCAKT mice (Figs 4F and Supplementary Figure S1A (see section on Supplementary materials given at the end of the article)). No difference in β-cell mass was measured between normal chow-fed groups (Supplementary Figs S1B and S1C). Percent area and number of islets per pancreas section were unchanged between HFFD-TAM-KMCAKT and HFFD-CO-KMCAKT mice (Supplementary Figs S1D, S1E and S1F). The alpha cell-to-beta cell ratio was also unchanged (Supplementary Fig. S1G). Together, these findings demonstrate that tamoxifen-induced mitochondrial Akt1 activation in kidney proximal tubules increased insulin secretion under metabolic stress.

To confirm that improved whole-body glucose metabolism was mediated by enhanced pancreatic insulin secretion, hyperglycemic clamp measurements were performed. During the 2-h hyperglycemic clamp, both HFFD-CO-KMCAKT mice and HFFD-TAM-KMCAKT mice achieved and maintained the target hyperglycemic plateau (Fig. 4G and H). However, HFFD-TAM-KMCAKT mice showed a marked increase in insulin secretion. Plasma insulin levels were significantly higher in HFFD-TAM-KMCAKT mice at early time points (15 and 30 min) and remained elevated during the sustained phase of the clamp (60, 90, and 120 min) as compared to HFFD-CO-KMCAKT mice (Fig. 4I), demonstrating enhanced glucose-stimulated insulin secretion and improved responsiveness to hyperglycemia. A similar pattern was observed for plasma C-peptide levels (Fig. 4J), indicating a robust increase in endogenous insulin secretion. Despite the enhancement of insulin and C-peptide release, early-phase blood glucose concentrations (15–40 min) were higher in HFFD-TAM-KMCAKT mice before stabilizing to levels similar to HFFD-CO-KMCAKT mice during the later phase of the clamp (Fig. 4H). The glucose infusion rate was comparable between the two groups, suggesting that whole-body insulin sensitivity remained unchanged despite enhanced insulin secretion (Fig. 4G).

## Discussion

DN remains a leading cause of CKD and subsequent ESRD worldwide (30, 31) despite advances in glucose-lowering and renoprotective therapies (32). In the present study, using a tamoxifen-inducible, renal tubule-specific mitochondria-targeted constitutively active Akt1 mouse model, we demonstrate that selective activation of mitochondrial Akt1 in renal proximal tubules confers robust protection against diet-induced DN and unexpectedly exerts systemic metabolic benefits by enhancing β-cell function without altering peripheral insulin sensitivity, body composition, or lipid metabolism. The presence of type 2 diabetes (T2D) and CKD is associated with a high future risk of CVDs that is similar to patients with prior myocardial infarction in comparison with diabetic patients without CKD (33). Moreover, CKD and CVD are very common in diabetic patients. A study among over 500,000 adults with T2D demonstrated that less than 10% had diabetes without accompanying CKD or CVD (34). Indeed, CKD and CVD exhibit a bidirectional relationship, where dysfunction in one organ worsens the other, leading to increased morbidity and mortality in individuals with T2D (35). Our findings suggest a cross talk between renal tubules and systemic metabolic homeostasis in an experimental model of diet-induced metabolic syndrome, which could be a new direction to uncover the complex interplays of the cardiovascular–kidney–metabolic syndrome.

### Renoprotective role of mitochondrial Akt1 against diet-induced diabetic nephropathy

Consistent with prior reports (36, 37), chronic exposure to high-fat and fructose feeding successfully induced obesity, insulin resistance, and hyperglycemia. With respect to kidney function, long-term HFFD resulted in albuminuria, renal fibrosis, and mesangial expansion, recapitulating key pathological features observed in human DN (10, 38, 39). Thus, our diet-induced mouse model can be used as an experimental model to study DN (36).

HFFD-induced diabetic mice demonstrated a significant elevation of plasma markers of kidney dysfunction, BUN (40), and transforming growth factor beta (TGFβ1) (41, 42, 43, 44), as well as an increase in renal TGFβ1 (45, 46) and alpha-smooth muscle actin (αSMA) (47) protein expression. TGFβ1 is a key profibrotic cytokine underlying the development of renal fibrosis in CKDs, including DN (48, 49). TGFβ1 induces epithelial–mesenchymal transition (50, 51), promotes tubular hypertrophy (52), and contributes to glomerular injuries (53). αSMA is a marker of activated myofibroblasts (54), which plays a critical role in the pathogenesis of renal fibrosis in CKD (55). Upon kidney injury, resident fibroblasts and pericytes differentiate into αSMA-positive myofibroblasts, which produce excessive extracellular matrix proteins, leading to scarring and tissue remodeling (56). In our study, tamoxifen-injected KMCAKT mice with activated mitochondrial Akt1 exhibited lower plasma concentrations of TGFβ1, along with lower expression of renal TGFβ1 and αSMA. Histological analysis of kidney tissue sections from diabetic mice revealed a significant increase in collagen deposition, glomerular basement membrane thickening, and tubular injury, confirming progressive renal structural damage and glomerulosclerosis, characteristic features of DN (10). These histological features were attenuated in TAM-KMCAKT mice. Renal tubular mitochondrial Akt1 likely limited glomerulotubular damage by attenuating profibrotic signaling cascades in diabetes. These data support the renoprotective role of tubular mitochondrial Akt1 activation in slowing or reversing progression of DN. Furthermore, these findings corroborate the notion that mitochondrial dysfunction is a major cause of tubular injury and subsequent progression of DN across multiple animal models (17, 57, 58, 59).

Akt1 signaling has long been recognized as a critical regulator of cell survival and metabolic adaptation in the kidney (23, 60). Our previous work demonstrated that mitochondrial translocation of Akt1 is an adaptive response to ischemia–reperfusion injury that preserves oxidative phosphorylation and limits mitochondrial ROS accumulation during acute kidney injury. Activation of tubular mitochondrial Akt1 delayed the development of CKD after acute ischemia–reperfusion injury, whereas inhibition exacerbated kidney injury (26). Consistent with this concept, a recent study showed that metabolic stress induces endogenous activated Akt1 translocation to proximal tubular mitochondria as an adaptive response in high-fat-diet and *db/db* mouse models, while blockade of this translocation reduced renal cell viability during high-fat exposure *in vitro* (61). Together with our gain-of-function findings, these results identify proximal tubular mitochondrial Akt1 activation as a critical adaptive and renoprotective signaling node that mitigates kidney tissue injury and preserves renal metabolic function. This observation is particularly relevant given the growing evidence that renal proximal tubule dysfunction is not merely a consequence of glomerular insult but may instead serve as a primary driver of DN progression (62, 63, 64).

### Metabolic role of tubular mitochondrial Akt1 in kidney–pancreas cross talk

Interestingly, tubular mitochondrial Akt1 activation also improved systemic glucose homeostasis. Tamoxifen-induced mitochondrial Akt1 activation in HFFD-fed KMCAKT mice resulted in lower fasting blood glucose and improved glucose tolerance when compared to HFFD-fed CO-KMCAKT controls, despite no significant changes in total body weight, body fat mass, food/water intake, or insulin sensitivity. Enhanced basal HOMA-β (homeostasis model assessment of beta cell function) (27) and higher glucose-stimulated insulin secretion during OGTTs suggest improved pancreatic insulin secretion (65). Notably, the increase in insulin secretion was associated with an increased beta cell mass without a change in the alpha cell-to-beta cell ratio. Hyperglycemic clamp measurements, the gold standard of measuring insulin secretion *in vivo* (66), confirmed enhanced insulin and C-peptide secretion during both early and late phases of glucose stimulation. Diet-induced insulin resistance (HOMA-IR) (27, 65, 67) was comparable among HFFD-fed KMCAKT mice regardless of mitochondrial Akt1 activation, reinforcing the concept that tubular mitochondrial Akt1 modulation of insulin secretion was independent of insulin sensitivity. Our data suggest a novel renal tubular mitochondria–pancreas cross talk. Since β-cell dysfunction is a critical determinant of glycemic homeostasis in type 2 diabetes (68, 69, 70), the recognition of tubular mitochondria–pancreas cross talk may have important implications for further understanding the dysregulation of glucose metabolism in diabetes.

The mechanism by which tubular mitochondrial Akt1 modulates pancreatic output remains unknown. The kidney is increasingly recognized as an endocrine and metabolic organ capable of influencing systemic glucose metabolism through gluconeogenesis, sodium–glucose co-transport system, inflammatory signaling, and mitochondrial-derived metabolites (63, 71, 72, 73). Improved tubular mitochondrial function may reduce renal oxidative stress and inflammation.

Additionally, reduced glucotoxicity may alleviate β-cell stress and promote β-cell health, thereby creating a more favorable systemic condition for insulin secretion. Kidney–pancreas cross talk may also involve a circulating kidney-derived soluble factor that directly modulates β-cell function or improves systemic metabolic/inflammatory stress to reduce renal injury. Further studies are necessary to elucidate the mechanistic link between tubular mitochondrial Akt1 and the pancreas. Our findings support a new paradigm in which renal mitochondrial health may influence whole-body metabolic homeostasis, extending beyond its traditional roles in renal function and blood pressure regulation (74). This concept is consistent with recent studies demonstrating bidirectional communication between kidney function and systemic glucose homeostasis and energy metabolism (75).

### Limitations

Whereas our KMCAKT model provides compartment-specific renoprotective effects of mitochondrial Akt1 activation, other studies have reported that sustained cytoplasmic Akt1 activation has been linked to maladaptive pathways, including fibroblast proliferation, extracellular matrix deposition, and tubular apoptosis, which contribute to renal fibrosis (76). Further studies should be performed to delineate the spatial compartmental coordination of overall Akt1 signaling effects in renal tubules. Second, the molecular mediators downstream of renal tubular mitochondrial Akt1 activation remain to be determined and warrant future investigation. Third, only male mice were used in the present study. Therefore, potential sex-dependent differences in proximal tubular mitochondrial Akt1 signaling, diabetic kidney injury, and systemic metabolic responses have not been assessed. Finally, the role of tubular mitochondrial Akt1 in human metabolic syndrome requires validation with properly designed human studies.

## Conclusion

This study identifies renal proximal tubular mitochondrial Akt1 as an important regulator of both kidney function and, remarkably, systemic glucose metabolism in diet-induced diabetes. Activation of tubular mitochondrial Akt1 not only protects against DN progression but also enhances insulin secretion without altering insulin sensitivity or body composition. These findings reveal a previously unrecognized kidney–pancreas cross talk mediated by mitochondrial Akt1 signaling. If this paradigm can be confirmed in human DN, targeting renal mitochondrial Akt1 signaling in proximal tubules may represent a novel opportunity to develop therapeutic strategies for the prevention and management of DN and the regulation of systemic glucose homeostasis.

## Supplementary materials



## Declaration of interest

The authors declare that there is no conflict of interest that could be perceived as prejudicing the impartiality of the work reported.

## Funding

This work was supported by the National Institutes of Health (R01HL096987) and Ko Family Foundation (to PHW).

## Author contribution statement

PHW conceived the research idea and provided overall supervision. ESBS and PHW designed the methodology and experiments. ESBS and EBS conducted experiments. PTF contributed to data interpretation. The manuscript was composed by ESBS, APT, and PHW, with inputs from PTF.

## AI disclosure

No generative artificial intelligence (AI) tools were used in the preparation of the written or visual content of this manuscript.

## References

[1] Tuttle KR, Bakris GL, Bilous RW, et al. Diabetic kidney disease: a report from an ADA consensus conference. Diabetes Care 2014 37 2864–2883. (10.2337/dc14-1296) PMC4170131 25249672

[2] Koye DN, Shaw JE, Reid CM, et al. Incidence of chronic kidney disease among people with diabetes: a systematic review of observational studies. Diabet Med 2017 34 887–901. (10.1111/dme.13324) 28164387

[3] Palsson R & Patel UD. Cardiovascular complications of diabetic kidney disease. Adv Chronic Kidney Dis 2014 21 273–280. (10.1053/j.ackd.2014.03.003) PMC4045477 24780455

[4] Saeedi P, Petersohn I, Salpea P, et al. Global and regional diabetes prevalence estimates for 2019 and projections for 2030 and 2045: results from the International Diabetes Federation Diabetes Atlas, 9(th) edition. Diabetes Res Clin Pract 2019 157 107843. (10.1016/j.diabres.2019.107843) 31518657

[5] Ogurtsova K, da Rocha Fernandes JD, Huang Y, et al. IDF Diabetes Atlas: global estimates for the prevalence of diabetes for 2015 and 2040. Diabetes Res Clin Pract 2017 128 40–50. (10.1016/j.diabres.2017.03.024) 28437734

[6] Collins AJ, Foley RN, Chavers B, et al. US renal data system 2013 annual data report. Am J Kidney Dis 2014 63 (Supplement 1) A7. (10.1053/j.ajkd.2013.11.001) 24360288

[7] Mann JFE, Orsted DD & Buse JB. Liraglutide and renal outcomes in type 2 diabetes. N Engl J Med 2017 377 2197–2198. 10.1056/NEJMc1713042 29171823

[8] Bennett WL, Maruthur NM, Singh S, et al. Comparative effectiveness and safety of medications for type 2 diabetes: an update including new drugs and 2-drug combinations. Ann Intern Med 2011 154 602–613. (10.7326/0003-4819-154-9-201105030-00336) PMC3733115 21403054

[9] Adler AI, Stevens RJ, Manley SE, et al. Development and progression of nephropathy in type 2 diabetes: the United Kingdom Prospective Diabetes Study (UKPDS 64). Kidney Int 2003 63 225–232. (10.1046/j.1523-1755.2003.00712.x) 12472787

[10] Tervaert TW, Mooyaart AL, Amann K, et al. Pathologic classification of diabetic nephropathy. J Am Soc Nephrol 2010 21 556–563. (10.1681/asn.2010010010) 20167701

[11] Takasu M, Kishi S, Nagasu H, et al. The role of mitochondria in diabetic kidney disease and potential therapeutic targets. Kidney Int Rep 2025 10 328–342. (10.1016/j.ekir.2024.10.035) PMC11843125 39990900

[12] Coughlan MT, Nguyen TV, Penfold SA, et al. Mapping time-course mitochondrial adaptations in the kidney in experimental diabetes. Clin Sci (Lond) 2016 130 711–720. (10.1042/cs20150838) 26831938

[13] Ha H, Kim C, Son Y, et al. DNA damage in the kidneys of diabetic rats exhibiting microalbuminuria. Free Radic Biol Med 1994 16 271–274. (10.1016/0891-5849(94)90152-x) 8005523

[14] Coughlan MT & Sharma K. Challenging the dogma of mitochondrial reactive oxygen species overproduction in diabetic kidney disease. Kidney Int 2016 90 272–279. (10.1016/j.kint.2016.02.043) 27217197

[15] Kang HM, Ahn SH, Choi P, et al. Defective fatty acid oxidation in renal tubular epithelial cells has a key role in kidney fibrosis development. Nat Med 2015 21 37–46. (10.1038/nm.3762) PMC4444078 25419705

[16] Ahmad AA, Draves SO & Rosca M. Mitochondria in diabetic kidney disease. Cells 2021 10 2945. (10.3390/cells10112945) PMC8616075 34831168

[17] Forbes JM & Thorburn DR. Mitochondrial dysfunction in diabetic kidney disease. Nat Rev Nephrol 2018 14 291–312. (10.1038/nrneph.2018.9) 29456246

[18] Huang C, Zhang Y, Kelly DJ, et al. Thioredoxin interacting protein (TXNIP) regulates tubular autophagy and mitophagy in diabetic nephropathy through the mTOR signaling pathway. Sci Rep 2016 6 29196. (10.1038/srep29196) PMC4933928 27381856

[19] Huang C, Lin MZ, Cheng D, et al. Thioredoxin-interacting protein mediates dysfunction of tubular autophagy in diabetic kidneys through inhibiting autophagic flux. Lab Invest 2014 94 309–320. (10.1038/labinvest.2014.2) 24492284

[20] Li W, Du M, Wang Q, et al. FoxO1 promotes mitophagy in the podocytes of diabetic Male mice via the PINK1/Parkin pathway. Endocrinology 2017 158 2155–2167. (10.1210/en.2016-1970) 28505239

[21] Badal SS & Danesh FR. New insights into molecular mechanisms of diabetic kidney disease. Am J Kidney Dis 2014 63 (Supplement 2) S63–S83. (10.1053/j.ajkd.2013.10.047) PMC3932114 24461730

[22] Sharma K, Karl B, Mathew AV, et al. Metabolomics reveals signature of mitochondrial dysfunction in diabetic kidney disease. J Am Soc Nephrol 2013 24 1901–1912. (10.1681/asn.2013020126) PMC3810086 23949796

[23] Bijur GN & Jope RS. Rapid accumulation of Akt in mitochondria following phosphatidylinositol 3-kinase activation. J Neurochem 2003 87 1427–1435. (10.1046/j.1471-4159.2003.02113.x) PMC2040497 14713298

[24] Chen YH, Ta AP, Chen Y, et al. Dual roles of myocardial mitochondrial AKT on diabetic cardiomyopathy and whole body metabolism. Cardiovasc Diabetol 2023 22 294. (10.1186/s12933-023-02020-1) PMC10612246 37891673

[25] Larsson L. The ultrastructure of the developing proximal tubule in the rat kidney. J Ultrastruct Res 1975 51 119–139. (10.1016/s0022-5320(75)80013-x) 1127791

[26] Lin HY, Chen Y, Chen YH, et al. Tubular mitochondrial AKT1 is activated during ischemia reperfusion injury and has a critical role in predisposition to chronic kidney disease. Kidney Int 2021 99 870–884. (10.1016/j.kint.2020.10.038) PMC7987863 33316281

[27] Matthews DR, Hosker JP, Rudenski AS, et al. Homeostasis model assessment: insulin resistance and beta-cell function from fasting plasma glucose and insulin concentrations in man. Diabetologia 1985 28 412–419. (10.1007/bf00280883) 3899825

[28] Nir T, Melton DA & Dor Y. Recovery from diabetes in mice by beta cell regeneration. J Clin Investig 2007 117 2553–2561. (10.1172/jci32959) PMC1957545 17786244

[29] Ayala JE, Bracy DP, McGuinness OP, et al. Considerations in the design of hyperinsulinemic-euglycemic clamps in the conscious mouse. Diabetes 2006 55 390–397. (10.2337/diabetes.55.02.06.db05-0686) 16443772

[30] Saran R, Li Y, Robinson B, et al. US renal data system 2015 annual data report: epidemiology of kidney disease in the United States. Am J Kidney Dis 2016 67 (Supplement 1) S1–S305. (10.1053/j.ajkd.2015.12.014) PMC6643990 26925525

[31] Papademetriou V, Lovato L, Doumas M, et al. Chronic kidney disease and intensive glycemic control increase cardiovascular risk in patients with type 2 diabetes. Kidney Int 2015 87 649–659. (10.1038/ki.2014.296) 25229335

[32] Mlynarska E, Bulawska D, Czarnik W, et al. Novel insights into diabetic kidney disease. Int J Mol Sci 2024 25 10222. (10.3390/ijms251810222) PMC11432709 39337706

[33] Tonelli M, Muntner P, Lloyd A, et al. Risk of coronary events in people with chronic kidney disease compared with those with diabetes: a population-level cohort study. Lancet 2012 380 807–814. (10.1016/s0140-6736(12)60572-8) 22717317

[34] Arnold SV, Kosiborod M, Wang J, et al. Burden of cardio-renal-metabolic conditions in adults with type 2 diabetes within the Diabetes Collaborative Registry. Diabetes Obes Metab 2018 20 2000–2003. (10.1111/dom.13303) 29577540

[35] Ronco C, Haapio M, House AA, et al. Cardiorenal syndrome. J Am Coll Cardiol 2008 52 1527–1539. (10.1016/j.jacc.2008.07.051) 19007588

[36] Dissard R, Klein J, Caubet C, et al. Long term metabolic syndrome induced by a high fat high fructose diet leads to minimal renal injury in C57BL/6 mice. PLoS One 2013 8 e76703. (10.1371/journal.pone.0076703) PMC3789664 24098551

[37] Panchal SK, Poudyal H, Iyer A, et al. High-carbohydrate, high-fat diet-induced metabolic syndrome and cardiovascular remodeling in rats. J Cardiovasc Pharmacol 2011 57 611–624. (10.1097/fjc.0b013e3181feb90a) 21572266

[38] Dalla VM, Saller A, Bortoloso E, et al. Structural involvement in type 1 and type 2 diabetic nephropathy. Diabetes Metab 2000 26 (Supplement 4) 8–14. 10922968

[39] Bakris GL & Molitch M. Microalbuminuria as a risk predictor in diabetes: the continuing saga. Diabetes Care 2014 37 867–875. (10.2337/dc13-1870) 24558077

[40] Xie Y, Bowe B, Li T, et al. Higher blood urea nitrogen is associated with increased risk of incident diabetes mellitus. Kidney Int 2018 93 741–752. (10.1016/j.kint.2017.08.033) 29241622

[41] Azar ST, Salti I, Zantout MS, et al. Alterations in plasma transforming growth factor beta in normoalbuminuric type 1 and type 2 diabetic patients. J Clin Endocrinol Metab 2000 85 4680–4682. (10.1210/jcem.85.12.7073) 11134127

[42] Shukla A, Kare K, Banerjee BD, et al. Study of serum transforming growth factor-beta 1 (TGF-β1) levels in type 2 diabetes mellitus patients with nephropathy. Biomed Res 2018 29 3213–3218. (10.4066/biomedicalresearch.29-18-950)

[43] Pertseva N, Borysova I & Chub D. TGF‐β1 and VCAM-1 serum concentrations as diagnostic biomarkers of diabetic kidney disease progression. Rom J Diabetes Nutr Metab Dis 2019 26 169–175. (10.2478/rjdnmd-2019-0018)

[44] Abbas HS, Nada SZ & Fadhel AA. Assessment of transforming growth factor-beta (TGF-β) and albumin to creatinine ratio (ACR) in patients with type 2 diabetic nephropathy. World J Pharm Pharm Sci 2022 11 88–103. (10.20959/wjpps20228-22902)

[45] Chen S, Jim B & Ziyadeh FN. Diabetic nephropathy and transforming growth factor-beta: transforming our view of glomerulosclerosis and fibrosis build-up. Semin Nephrol 2003 23 532–543. (10.1053/s0270-9295(03)00132-3) 14631561

[46] Goldfarb S & Ziyadeh FN. TGF-beta: a crucial component of the pathogenesis of diabetic nephropathy. Trans Am Clin Climatol Assoc 2001 112 27–32. PMC2194397 11413780

[47] Sebe A, Leivonen SK, Fintha A, et al. Transforming growth factor-beta-induced alpha-smooth muscle cell actin expression in renal proximal tubular cells is regulated by p38beta mitogen-activated protein kinase, extracellular signal-regulated protein kinase1,2 and the Smad signalling during epithelial-myofibroblast transdifferentiation. Nephrol Dial Transplant 2008 23 1537–1545. (10.1093/ndt/gfm789) 18192325

[48] Ziyadeh F. Evidence for the involvement of transforming growth factor-β in the pathogenesis of diabetic kidney disease: are Koch's postulates fulfilled? Curr Pract Med 1995 1 87–89.

[49] Ziyadeh FN & Han DC. Involvement of transforming growth factor-beta and its receptors in the pathogenesis of diabetic nephrology. Kidney Int Suppl 1997 60 S7–S11. 9285895

[50] Hills CE & Squires PE. TGF-beta1-induced epithelial-to-mesenchymal transition and therapeutic intervention in diabetic nephropathy. Am J Nephrol 2010 31 68–74. (10.1159/000256659) 19887790

[51] Forino M, Torregrossa R, Ceol M, et al. TGFbeta1 induces epithelial-mesenchymal transition, but not myofibroblast transdifferentiation of human kidney tubular epithelial cells in primary culture. Int J Exp Pathol 2006 87 197–208. (10.1111/j.1365-2613.2006.00479.x) PMC2517360 16709228

[52] Chen S, Hoffman BB, Lee JS, et al. Cultured tubule cells from TGF-beta1 null mice exhibit impaired hypertrophy and fibronectin expression in high glucose. Kidney Int 2004 65 1191–1204. (10.1111/j.1523-1755.2004.00492.x) 15086458

[53] Rani P, Koulmane Laxminarayana SL, Swaminathan SM, et al. TGF-beta: elusive target in diabetic kidney disease. Ren Fail 2025 47 2483990. (10.1080/0886022x.2025.2483990) PMC11980245 40180324

[54] Hinz B, Phan SH, Thannickal VJ, et al. The myofibroblast: one function, multiple origins. Am J Pathol 2007 170 1807–1816. (10.2353/ajpath.2007.070112) PMC1899462 17525249

[55] Loeffler I & Wolf G. Epithelial-to-mesenchymal transition in diabetic nephropathy: fact or fiction? Cells 2015 4 631–652. (10.3390/cells4040631) PMC4695850 26473930

[56] Liu Y. Cellular and molecular mechanisms of renal fibrosis. Nat Rev Nephrol 2011 7 684–696. (10.1038/nrneph.2011.149) PMC4520424 22009250

[57] Wei PZ & Szeto CC. Mitochondrial dysfunction in diabetic kidney disease. Clin Chim Acta 2019 496 108–116. (10.1016/j.cca.2019.07.005) 31276635

[58] Dugan LL, You YH, Ali SS, et al. AMPK dysregulation promotes diabetes-related reduction of superoxide and mitochondrial function. J Clin Investig 2013 123 4888–4899. (10.1172/jci66218) PMC3809777 24135141

[59] Flemming N, Pernoud L, Forbes J, et al. Mitochondrial dysfunction in individuals with diabetic kidney disease: a systematic review. Cells 2022 11 2481. (10.3390/cells11162481) PMC9406893 36010558

[60] Datta SR, Dudek H, Tao X, et al. Akt phosphorylation of BAD couples survival signals to the cell-intrinsic death machinery. Cell 1997 91 231–241. (10.1016/s0092-8674(00)80405-5) 9346240

[61] Lin HY, Chen IY, Wang TM, et al. The role of mitochondrial AKT1 signaling in renal tubular injury of metabolic syndrome. Kidney Int Rep 2025 10 906–920. (10.1016/j.ekir.2024.12.021) PMC11993225 40225378

[62] Gilbert RE. Proximal tubulopathy: prime mover and key therapeutic target in diabetic kidney disease. Diabetes 2017 66 791–800. (10.2337/db16-0796) 28325740

[63] Vallon V & Thomson SC. The tubular hypothesis of nephron filtration and diabetic kidney disease. Nat Rev Nephrol 2020 16 317–336. (10.1038/s41581-020-0256-y) PMC7242158 32152499

[64] Vallon V & Thomson SC. Renal function in diabetic disease models: the tubular system in the pathophysiology of the diabetic kidney. Annu Rev Physiol 2012 74 351–375. (10.1146/annurev-physiol-020911-153333) PMC3807782 22335797

[65] Ahren B & Pacini G. Importance of quantifying insulin secretion in relation to insulin sensitivity to accurately assess beta cell function in clinical studies. Eur J Endocrinol 2004 150 97–104. (10.1530/eje.0.1500097) 14763905

[66] DeFronzo RA, Tobin JD & Andres R. Glucose clamp technique: a method for quantifying insulin secretion and resistance. Am J Physiol 1979 237 E214–E223. (10.1152/ajpendo.1979.237.3.e214) 382871

[67] Winzell MS & Ahren B. The high-fat diet-fed mouse: a model for studying mechanisms and treatment of impaired glucose tolerance and type 2 diabetes. Diabetes 2004 53 (Supplement 3) S215–S219. 10.2337/diabetes.53.suppl_3.s215 15561913

[68] Weir GC & Bonner-Weir S. Five stages of evolving beta-cell dysfunction during progression to diabetes. Diabetes 2004 53 (Supplement 3) S16–S21. (10.2337/diabetes.53.suppl_3.s16) 15561905

[69] Prentki M & Nolan CJ. Islet beta cell failure in type 2 diabetes. J Clin Investig 2006 116 1802–1812. (10.1172/jci29103) PMC1483155 16823478

[70] Ogihara T & Mirmira RG. An islet in distress: beta cell failure in type 2 diabetes. J Diabetes Investig 2010 1 123–133. (10.1111/j.2040-1124.2010.00021.x) PMC4008003 24843420

[71] Gerich JE. Role of the kidney in normal glucose homeostasis and in the hyperglycaemia of diabetes mellitus: therapeutic implications. Diabet Med 2010 27 136–142. (10.1111/j.1464-5491.2009.02894.x) PMC4232006 20546255

[72] DeFronzo RA, Davidson JA & Del Prato S. The role of the kidneys in glucose homeostasis: a new path towards normalizing glycaemia. Diabetes Obes Metab 2012 14 5–14. (10.1111/j.1463-1326.2011.01511.x) 21955459

[73] Mather A & Pollock C. Glucose handling by the kidney. Kidney Int 2011 79(Supp. 120) S1–S6. (10.1038/ki.2010.509) 21358696

[74] Tian Z & Liang M. Renal metabolism and hypertension. Nat Commun 2021 12 963. (10.1038/s41467-021-21301-5) PMC7878744 33574248

[75] Kumar M, Dev S, Khalid MU, et al. The bidirectional link between diabetes and kidney disease: mechanisms and management. Cureus 2023 15 e45615. (10.7759/cureus.45615) PMC10588295 37868469

[76] Kim IY, Song SH, Seong EY, et al. Akt1 is involved in renal fibrosis and tubular apoptosis in a murine model of acute kidney injury-to-chronic kidney disease transition. Exp Cell Res 2023 424 113509. (10.1016/j.yexcr.2023.113509) 36773738

