# Supplementary material for: Dual roles of renal tubular mitochondrial Akt1 in protecting against diabetic nephropathy and improving body glucose metabolism
Source: J Endocrinol. 2026 Sep 15;270(3):e260143. doi: 10.1530/JOE-26-0143 (PMC13615847; doi:10.1530/JOE-26-0143)

Supplemental Figure 1

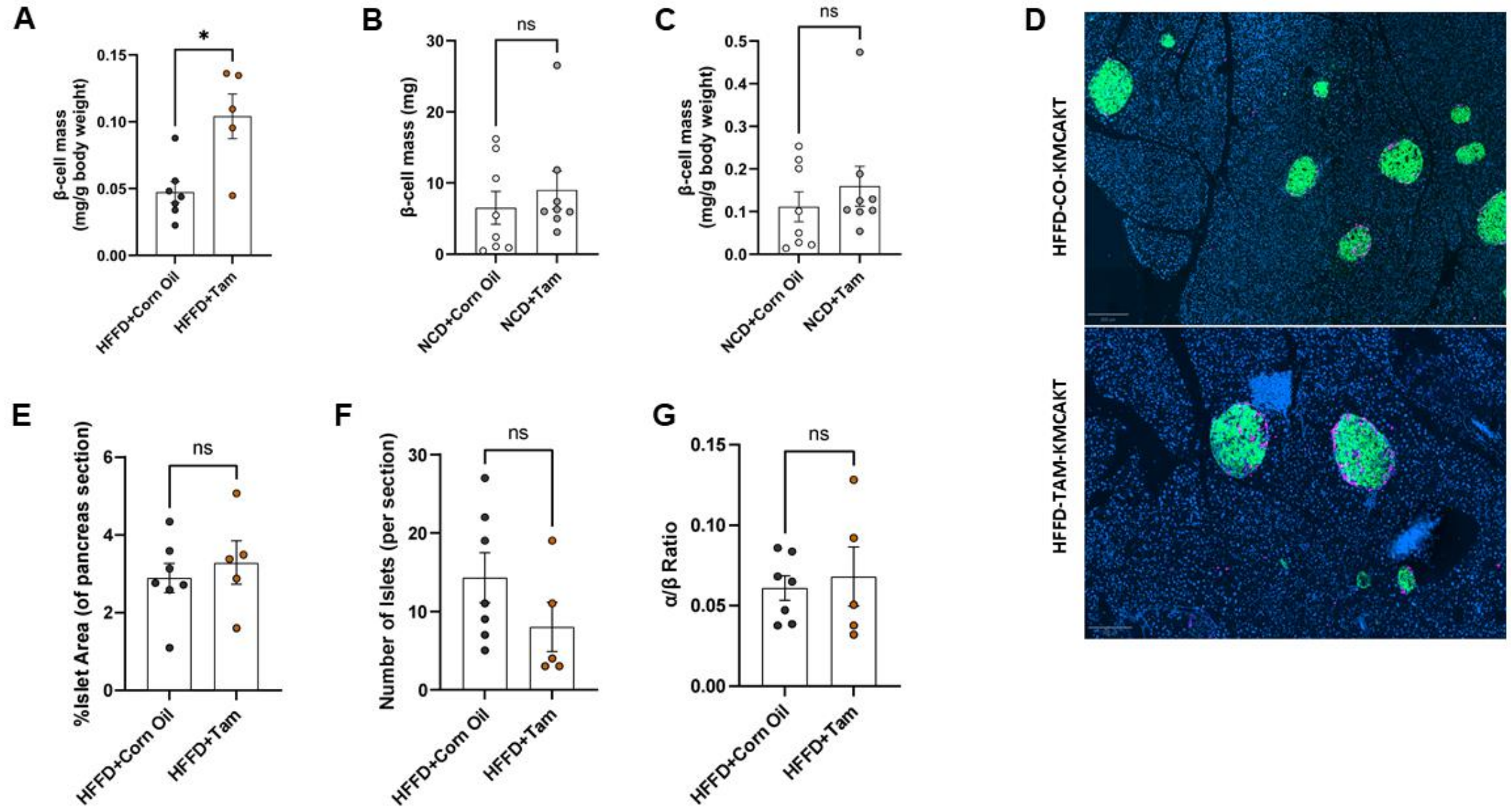

Supplement: Supplementary file 1 [file JOE-26-0143_supplementary_figure_1.pdf]
